# The current advances of lncRNAs in breast cancer immunobiology research

**DOI:** 10.3389/fimmu.2023.1194300

**Published:** 2023-06-05

**Authors:** Marco Antonio Fonseca-Montaño, Karla Itzel Vázquez-Santillán, Alfredo Hidalgo-Miranda

**Affiliations:** ^1^ Laboratorio de Genómica del Cáncer, Instituto Nacional de Medicina Genómica (INMEGEN), Mexico City, Mexico; ^2^ Programa de Doctorado, Posgrado en Ciencias Biológicas, Unidad de Posgrado, Universidad Nacional Autónoma de México (UNAM), Mexico City, Mexico; ^3^ Laboratorio de Epigenética, Instituto Nacional de Medicina Genómica (INMEGEN), Mexico City, Mexico

**Keywords:** breast cancer, immune cells, tumor immune microenvironment, lncRNAs, immune response, biomarker, prognosis

## Abstract

Breast cancer is the most frequently diagnosed malignancy and the leading cause of cancer-related death in women worldwide. Breast cancer development and progression are mainly associated with tumor-intrinsic alterations in diverse genes and signaling pathways and with tumor-extrinsic dysregulations linked to the tumor immune microenvironment. Significantly, abnormal expression of lncRNAs affects the tumor immune microenvironment characteristics and modulates the behavior of different cancer types, including breast cancer. In this review, we provide the current advances about the role of lncRNAs as tumor-intrinsic and tumor-extrinsic modulators of the antitumoral immune response and the immune microenvironment in breast cancer, as well as lncRNAs which are potential biomarkers of tumor immune microenvironment and clinicopathological characteristics in patients, suggesting that lncRNAs are potential targets for immunotherapy in breast cancer.

## 1 Introduction

Breast cancer (BC) is the most frequently diagnosed malignancy and the leading cause of cancer-related death in women worldwide (1, 2). BC is a multifactorial and heterogeneous disease that includes well-defined histological types and protein markers, such as estrogen receptor (ER), progesterone receptor (PR), human epidermal growth factor receptor 2 (HER2), and Ki-67 (1, 3–5). According to the PAM50 gene signature, BC is classified into Luminal A (LA), Luminal B (LB), HER2-enriched, and Basal-like (BL) subtypes. Remarkably, luminal BCs represent around 60 to 70% of diagnosed cases and are frequently associated with improved prognosis, in contrast to non-luminal subtypes (1, 6, 7). Understanding the alterations in specific genes and disrupted signaling pathways involved in BC is essential to unravel the underlying mechanisms of development and progression. In this regard, accumulated evidence has shown recurrent alterations in diverse genes (i.e., *TP53, ESR1, PIK3CA, PTEN, CDH1, GATA3, CCND1, FGFR1/2, ERBB2, CDKN2A/2B, MYC* and *BRCA1/2)* as well as dysregulations in various signaling pathways (i.e., hormone receptors, DNA damage repair, PI3K/AKT/mTOR, MAPK/ERK, TGF-β, NFκB, WNT/β-Catenin, Notch, Hippo, and SHH), which are associated with cell survival, proliferation, epithelial-mesenchymal transition (EMT), therapy resistance, immune evasion and tumor immune microenvironment (TIME) alterations in BC (1, 8–19).

The TIME consists of dynamic niches where cancer cells coexist and interact with diverse lymphoid (i.e., natural killer (NK) cells, B cells, CD4^+^ T cells, CD8^+^ T cells, regulatory T cells, and T follicular helper cells) and myeloid immune cell populations (i.e., dendritic cells (DCs), mast cells, myeloid-derived suppressor cells (MDSCs), M0, M1, M2 macrophages, and neutrophils), as well as with soluble factors secreted by these cells (i.e., cytokines and chemokines) in the extracellular matrix. Notably, the cancer genotype and phenotype have a crucial role in the TIME’s composition and functionality, varying depending on the cancer type and clinical stage. In this context, TIME is essential at primary, pre-metastatic, and metastatic sites (20–22). Therefore, the immune context in cancer is associated with prognosis and therapeutic efficacy in patients (23, 24). Previous articles have reviewed the cancer-immunity cycle and the cancer-immune set point for a better understanding of cancer immunobiology (25, 26). Remarkably, several studies have characterized and analyzed the composition and functionality of tumor-infiltrating immune cells (TIICs) across different BC subtypes, evidencing significant associations with prognosis in patients (27–34). Immunotherapies for BC treatment based on CAR T cells, CAR NK cells, immune-checkpoint (IC) inhibitors, cytokine modulation, chemotherapy drugs to induce immunogenic cell death, and personalized vaccines related to tumor-associated antigens are being tested in clinical trials (35). Despite these advances, there is still a lack of knowledge to understand BC immunobiology fully. A fascinating research field focused on long non-coding RNAs (lncRNAs) is being explored in this context.

LncRNAs are non-protein-coding transcripts of more than 200 nucleotides in length and are classified according to their location and orientation relative to protein-coding genes into sense, anti-sense, intronic, intergenic, and bidirectional (36, 37). Notably, lncRNAs are frequently transcribed by RNA polymerase II (Pol II) and III (Pol III). Pol II-transcribed lncRNAs are spliced, bear 7-methyl guanosine caps at the 5’ end, and have polyadenylated tails at the 3’ end. In contrast, Pol III-transcribed lncRNAs lack caps and poly-A tails. Remarkably, the expression of lncRNAs is lower compared to protein-coding genes. However, lncRNAs exhibit higher tissue and cell specificity, highlighting their regulatory roles (36, 37).

LncRNAs may act in the nucleus or cytoplasm cell fraction, exhibiting a wide range of functions. In a non-pathological context, lncRNAs have essential roles in diverse biological processes, such as regulation of gene expression, chromatin modification, genomic imprinting, and transcriptional and translational processing (36, 37). These functions are mainly achieved due to lncRNA may interact with diverse DNA elements (i.e., exons, introns, and promoters), RNA species (i.e., mRNAs, miRNAs, and other lncRNAs), proteins (i.e., related to epigenetic, transcriptional, translational processes and extracellular vesicles). Previous findings have demonstrated that dysregulation of lncRNAs is associated with cancer biology, evidencing that these molecules are critical modulators of cancer signaling pathways and may act as oncogenes and tumor suppressors, showing versatile and complex roles associated with diverse hallmarks of cancer (38–43). Notably, various reports have evidenced that functional mechanisms and dysregulations associated with various lncRNAs (i.e., SPRY4-IT1, DANCR, PVT1, TUSC8, ATV1, LINC00617, PICART1, APOC1P1-3, SERM, and SERT) are promoters of cell proliferation, invasion, migration, apoptosis, stemness, and drug resistance in BC (44). Particularly, recent investigations have demonstrated the importance of lncRNAs in BC immunobiology, showing that lncRNAs are essential players in the antitumoral immune response, immune evasion mechanisms, and composition and functionality of the TIME. Abnormal expression of lncRNAs has been shown to affect the immune phenotypes across different cancer types, including BC (45–48). In this review, we provide the current advances about the role of lncRNAs as tumor-intrinsic and tumor-extrinsic modulators of the antitumoral immune response and TIME in BC, as well as lncRNAs which are potential biomarkers of the TIME and clinicopathological characteristics in BC patients.

## 2 LncRNAs as tumor-intrinsic modulators of the antitumoral immune response in BC

Previous research demonstrated that metabolic changes, loss of antigenicity, upregulation of immune inhibitory factors, and alterations in the TIME are important tumor-intrinsic mechanisms of immune evasion and immunotherapy resistance across different cancer types (23, 49, 50). In addition, the dysregulation of oncogenic pathways, such as WNT/β-catenin, MYC, LKB1, PTEN, and TP53, have a crucial role in the promotion and suppression of local antitumor immune response, depending on the cell context and cancer type (8). Prior findings evidenced that *BRCA1, BRCA2*, and *TP53* mutations are associated with high mutational burden, neoantigen load, tumor-infiltrating lymphocyte density, high cytolytic activity, and improved prognosis in BC. Interestingly, the crosstalk between *BRCA1/BRCA2* alterations with NFkB, NOTCH, and PTEN signaling pathways hampers the immune response in BC (51–59). Remarkably, recent studies have evidenced that lncRNAs are critical regulators of cancer immunobiology (45–48). In this regard, the role of diverse lncRNAs as tumor-intrinsic modulators of BC immunobiology has been explored, identifying lncRNAs that function as promoters and suppressors of the antitumoral immune response.

### 2.1 Tumor-intrinsic lncRNAs as promoters of the antitumoral immune response in BC

**Figure 1:**
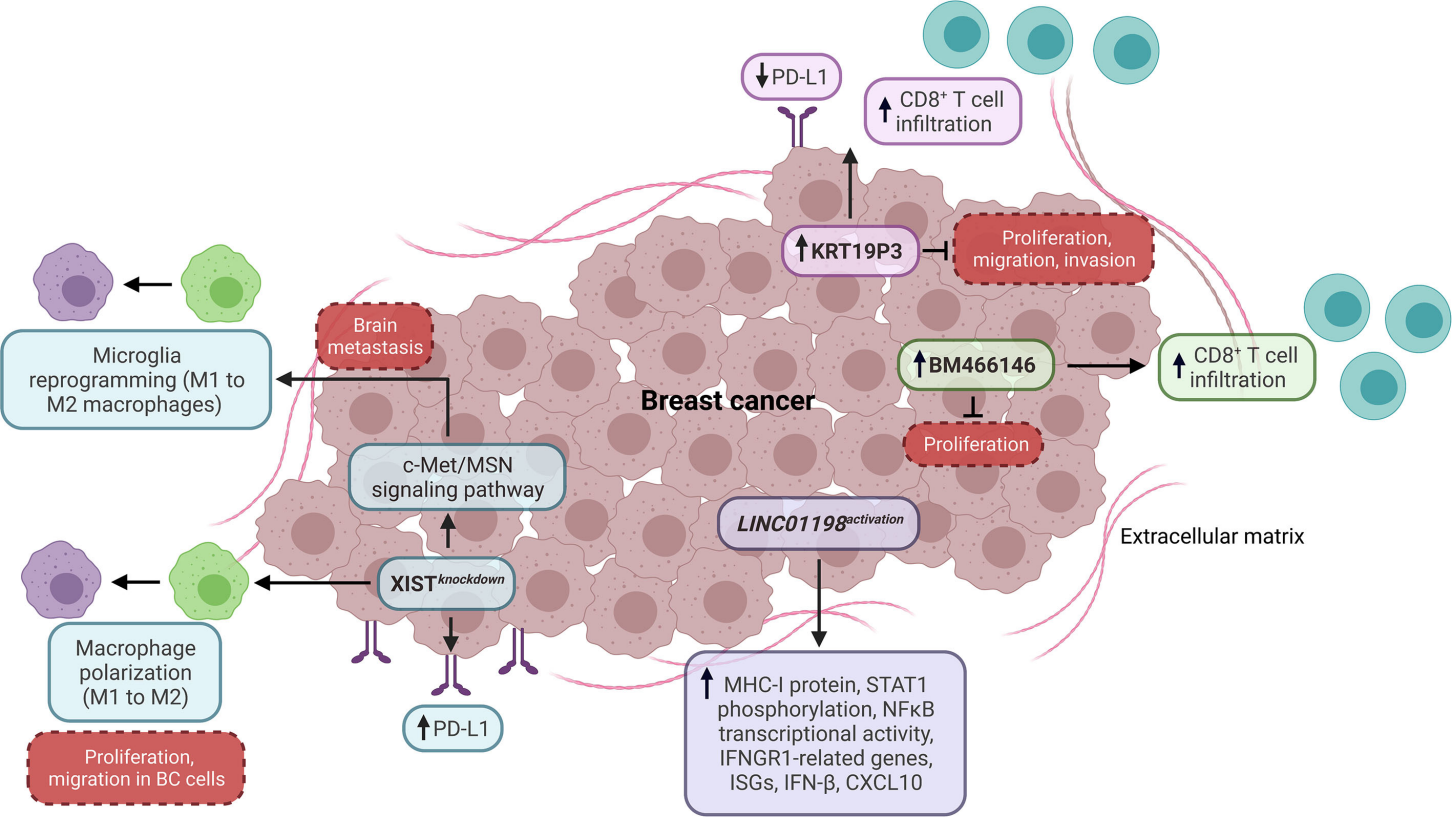
Impact of lncRNAs as tumor-intrinsic promoters of the immune response in BC. LncRNAs, including XIST, KRT19P3, BM466146, and LINC01198, function as tumor-intrinsic promoters of the immune response in BC, preventing PD-L1 expression, macrophage polarization, and promoting CD8^+^ T cell infiltration, as well as the expression of genes and proteins associated with antitumoral immune response. Dashed boxes in red denote cancer-related phenotypes. ISGs, interferon-stimulated genes.

Recent findings have evidenced the regulatory roles of lncRNAs as tumor-intrinsic promoters of the antitumoral immune response in BC. Salama et al. identified that triple-negative breast cancers (TNBC) exhibit a high expression of PD-L1 and a low expression of XIST. Notably, the XIST knockdown promotes an increased expression of PD-L1 in TNBC cells (60). In addition, Zhao et al. evidenced that the XIST knockdown promotes macrophage polarization from M1 to M2, supporting the proliferation and migration of BC cell lines (61). Another research demonstrated that the XIST loss upregulates the c-Met/MSN signaling pathway in TNBC, promoting brain metastasis. Specifically, the XIST loss promotes the microglia reprogramming from M1 to M2 macrophages by exosomal miR-503 releasing, STAT3, and NFκB pathways. Furthermore, BL and TNBC patients have a low expression of XIST, which is associated with poor metastasis-free survival (62). In addition, Hamed et al. showed that the oleuropein compound promotes the expression of XIST and the inhibition of miR194-5p/PD-L1 in TNBC, suggesting the feasibility of modulating the BC immunobiology by targeting lncRNAs and IC inhibitors (63). Overall, XIST is a positive regulator of the antitumoral immune response by preventing PD-L1 expression and M2 macrophage-related phenotypes in BC (**Figure 1**).

Another study showed that the high expression of KRT19P3 is related to the low expression of PD-L1 and high infiltration of CD8^+^ T cells in BC, indicating that this lncRNA might have an essential role in the T cell function through the PD-L1 modulation. Also, KRT19P3 decreases proliferation, migration, and invasion *in vitro* (64) (**Figure 1**). Otherwise, Beltran-Anaya et al. found that LncKLHDC7B is enriched in TNBC immunomodulatory subtype samples with high immunophenoscore values. The silencing of this lncRNA promotes cell migration and invasion while decreasing apoptosis *in vitro*. In addition, the low expression of LncKLHDC7B is associated with recurrence, metastatic events, and reduced survival in TNBC patients (65). In other research, Zhang et al. found that the expression of lncRNA BM466146 positively and negatively correlates with the infiltration level of CD8^+^ T cells and the Ki-67 index in BC patients, respectively. Particularly, BM466146 could upregulate the CXCL13 expression to recruit CD8^+^ T cells to the BC immune microenvironment. Also, the overexpression of BM466146 reduces the proliferation *in vitro*, while the high expression of this lncRNA is associated with increased overall survival (OS) in BC patients (66). In addition, an investigation based on an innovative CRISPR activation screening strategy showed that *LINC01198* is suppressed in diverse cancer types, including BC. Additional analyses demonstrated that IFNGR1-related genes, MHC-I protein expression, and STAT1 phosphorylation increase when *LINC01198* is activated in BC cells, while its inhibition decreases the expression of type I IFN pathway-related genes. Specifically, the activation of *LINC01198* promotes the expression of CXCL10, IFN-β, type I IFN receptors, interferon-stimulated genes (ISGs), and the transcriptional activity of NFκB (related to p65 component) *in vitro* (**Figure 1**). Equally important, the high expression of *LINC01198* is associated with a high score of CD8, IL-2, IL-8, and IL-12 immune signatures and improved OS in BC patients, indicating that *LINC01198* is a promoter of IFN-related antitumoral immune response (67).

### 2.2 Tumor-intrinsic lncRNAs as suppressors of the antitumoral immune response in BC

Different studies have shown the regulatory roles of lncRNAs as tumor-intrinsic suppressors of the antitumoral immune response in BC. In this context, Salama et al. demonstrated that the TSIX knockdown (a negative regulator of XIST) promotes a reduced expression of PD-L1 in TNBC cells. Moreover, TSIX is highly expressed in TNBC patients with high expression of PD-L1 (60). In another research, Samir et al. exhibited that the increased expression of MALAT1 and miR-182-5p positively modulates the PD-L1 expression through a negative and positive regulation of XIST and TSIX expression, respectively, promoting an immunosuppressive phenotype in TNBC (68). Recent findings showed that MALAT1 knockdown promotes the expression of stress-induced ligands (MICA and MICB) and the repression of immune checkpoints (PD-L1 and B7-H4) in TNBC cells. Also, the MALAT1 knockdown boosts the NK cells-mediated killing and CD8^+^ T cells-mediated cytotoxic activity *via* miR-34a and miR-17-5p, respectively, indicating that this lncRNA hampers the innate and adaptive immune response in TNBC (69). In another report, Xiping et al. found that MALAT1 knockdown decreases the expression of MYC oncogene and CD47 (a protein that binds to SIRPα and blocks the antigen uptake mediated by macrophages and DCs) in HER2+ and TNBC cells. In addition, the MALAT1 expression promotes proliferation and invasion *in vitro*, supporting the role of this lncRNA in the immune evasion of BC (70). A study showed that the methoxylated quercetin glycoside compound diminishes the MALAT1 expression, altering the immunogenic profile in BC (71). In addition, Wang et al. demonstrated that TINCR is a promoter of immune evasion in BC. Specifically, TINCR acts as a molecular sponge for miR-199a-5p and upregulates the USP20 stability through a ceRNA regulatory mechanism, promoting the upregulation of PD-L1 protein by inhibiting its ubiquitination. Additional analyses revealed that TINCR transcription is promoted through the activation of STAT1 signaling by IFNγ stimulation. Moreover, the TINCR knockdown reduces tumor growth, cell proliferation, migration, and invasion in BC (72).

**Figure 2:**
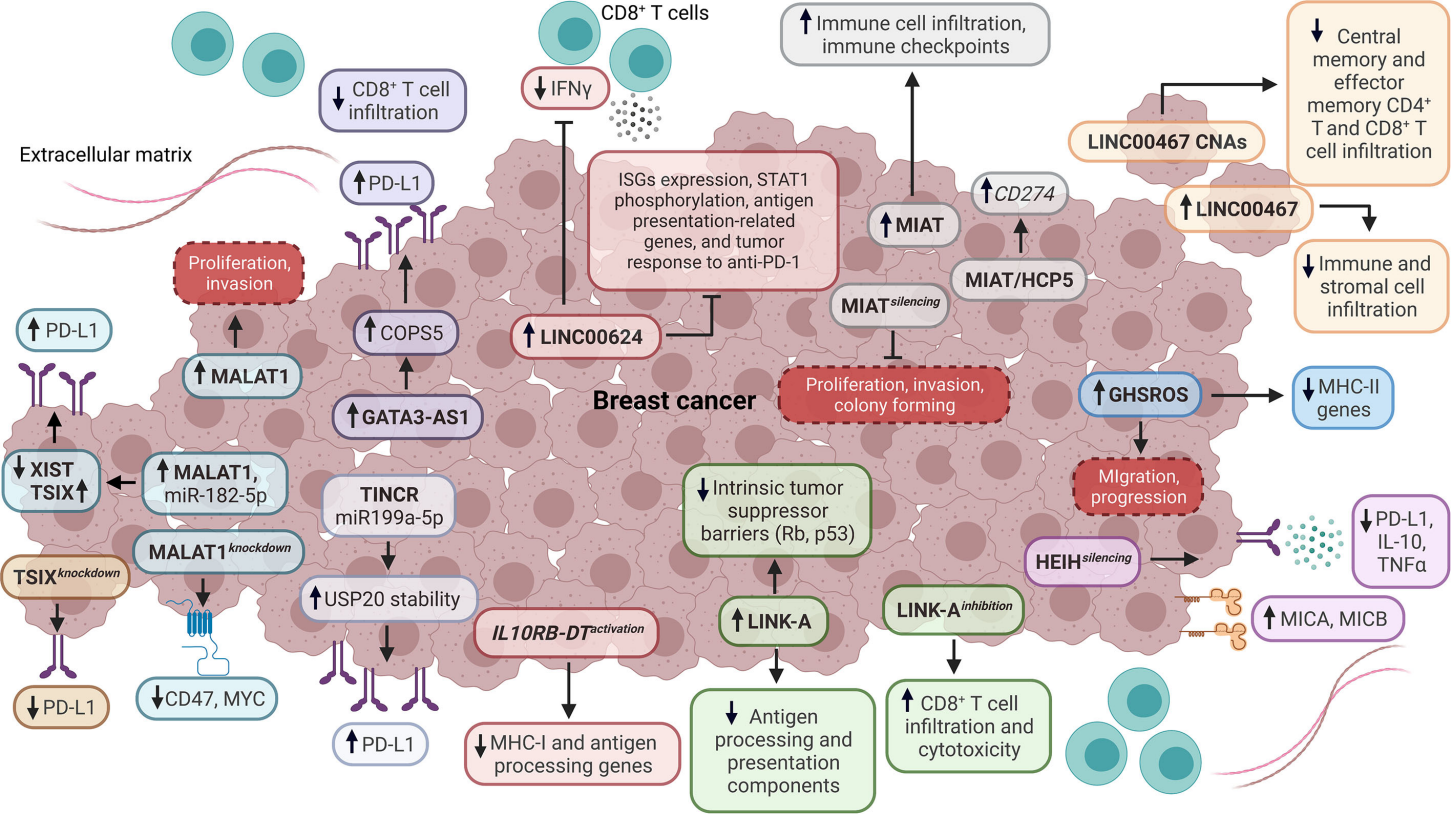
Impact of lncRNAs as tumor-intrinsic suppressors of the immune response in BC. Diverse lncRNAs, such as TSIX, TINCR, MALAT1, GATA3-AS1, LINC00624, IL10RB-DT, LINK-A, HEIH, GHSROS, LINC00467, MIAT, and HCP5 function as tumor-intrinsic suppressors of immune response in BC, dysregulating the expression of antigen processing and presentation components, interferon-stimulated genes (ISGs), immune checkpoints, cytokines, stress-induced ligands, STAT1 phosphorylation, tumor suppressor barriers, oncogenes, immunotherapy response, infiltration and functionality of tumor-infiltrating immune cells. Dashed boxes in red denote cancer-related phenotypes. CNAs, copy number amplifications.

On the other hand, a comprehensive investigation found that GATA3-AS1 expression enhances the PD-L1 protein levels and promotes cell proliferation and migration of TNBC cells. Particularly, GATA3-AS1 promotes the deubiquitination of PD-L1 protein through the upregulation of COPS5. Besides, the upregulation of GATA3-AS1 is related to a reduced percentage of CD8^+^ T cells in TNBC, and the high expression of this lncRNA is associated with reduced OS. In contrast, the high level of PD-L1 protein correlates with poor prognosis, large tumor size, and clinical stage in TNBC patients (73) (**Figure 2**). Another study demonstrated that HEIH is highly expressed in TNBC. At the same time, silencing this lncRNA reduces the expression of miR-939-5p, NOS2, decreases NO production and inhibits cell viability and migration *in vitro*. Moreover, the HEIH silencing increases the expression of MICA and MICB while decreasing the expression of PD-L1, IL-10, and TNFα, suggesting that HEIH significantly promotes immunosuppressive phenotypes in TNBC (74).

Hu et al. evidenced that LINK-A is upregulated in TNBC and negatively correlates with antigen-presenting cells and CD8^+^ T cell levels in BL BC. Particularly, LINK-A downregulates antigen processing and presentation components (i.e., TPSN, TAP1, TAP2, and CALR) and intrinsic tumor suppressor barriers (Rb and p53), which indicates that this lncRNA promotes tumor immune evasion (**Figure 2**). Likewise, TNBC patients who are responders to Pembrolizumab have a low expression of LINK-A and high infiltration of CD8^+^ T cells, in contrast to non-responders. Remarkably, LINK-A inhibition improves the CD8^+^/CD3^+^ T cell infiltration and cytotoxicity, indicating that this lncRNA might be a potential immunosuppressive biomarker and therapeutic target in TNBC patients (75). Wang et al. characterized the function of *IL10RB-DT* through a CRISPR activation screening. They found that the activation of this lncRNA inhibits the transcription of MHC-I and antigen-processing genes in BC cells. Equally important, the *IL10RB-DT* expression is associated with poor survival in BC patients (67). A study about the LINC00624 expression showed that this lncRNA negatively correlates with type I IFN and antigen processing and presentation pathways *in vitro*. Also, the overexpression of LINC00624 inhibits the ISGs expression and STAT1 phosphorylation *in vitro*. Additionally, IFNα induces the LINC00624 expression, suggesting that this lncRNA is an ISG that is a negative feedback modulator of the IFN signaling pathway (76) (**Figure 2**). Further analyses demonstrated that ADAR1 interacts with LINC00624, promoting A-to-I substitutions in this lncRNA *in vitro*, which increase after IFNα treatment. Interestingly, the function of the edited LINC00624 depends on ADAR1 to inhibit the IFN response and to promote Lapatinib and anti-HER2 treatment resistance in HER2+ BC cells. Also, tumor cells overexpressing LINC00624 co-cultured with CD8^+^ T cells inhibit IFNγ production *in vivo*. In contrast, the antigen presentation-related genes, ISGs, and tumor response to anti-PD-1 treatment are inhibited by LINC00624 *in vivo* (76) (**Figure 2**). This lncRNA is highly expressed in HER2+ BC patients with a non-pathological complete response, and the high expression of LINC00624 is associated with poor disease-free survival (DFS) (76).

Recent research evidenced that the lncRNA MIAT is co-expressed with different genes related to immune cells’ regulation, activation, and adhesion. BC patients with high expression of MIAT exhibit a high infiltration of CD8^+^ T cells, resting memory CD4^+^ T cells, activated memory CD4^+^ T cells, gamma-delta T cells, and M1 macrophages. In contrast, the infiltration of plasma cells, activated NK cells, monocytes, M2 macrophages, and activated mast cells are reduced (77). Furthermore, the MIAT expression positively correlates with IC genes like *PDCD1, CD274*, and *CTLA-4*, which are critical players in suppressing the antitumoral response mediated by T cells (**Figure 2**). Moreover, the high expression of MIAT is associated with clinical stage and lymph node metastasis in serum samples derived from BC patients. The high expression of MIAT is associated with reduced OS in BL BC. In contrast, the high expression of this lncRNA is associated with reduced post-progression survival in LA, LB, and HER2+ BC patients, which indicates a subtype-specific prognostic role of MIAT (77). Additional research confirmed that this lncRNA positively correlates with IC gene expression and its prognostic role associated with OS. Also, MIAT silencing reduces proliferation, colony forming, and invasion, while increasing TNBC cell apoptosis *in vivo*, indicating that MIAT is a promoter of immunosuppressive phenotypes in BC (78). A similar behavior was detected for lncRNA HCP5 in BC. Additional analyses showed that MIAT and HCP5 upregulate the expression of CD274 through a ceRNA mechanism, which involves miR-150-5p sponging in human cancer (78) (**Figure 2**). Interestingly, Wu et al. found that a ceRNA network composed of BTN3A1-has-miR-20b-5p-HCP5 could have a role in the interaction between BC cells and T cells *in vitro* (79). In this regard, different studies have indicated that ceRNA networks are composed of mRNAs-miRNAs-lncRNAs and are potential modulators of the TIME in BC (80–83).

Another study evidenced that the overexpression of lncRNA GHSROS induces the downregulation of MHC-II genes (*HLA-DRA, HLA-DPB1, HLA-DPA1*, and *HLA-DRB3*) in TNBC cells. In addition, the overexpression of GHSROS is associated with the downregulation of immune-related pathways, including antigen processing and presentation signaling. The expression of GHSROS promotes cell migration *in vitro* and progression *in vivo*, suggesting that this lncRNA is involved in BC immune evasion (84) (**Figure 2**). In another study, Bo et al. identified a high expression of LINC00467 in metastatic BC and circulating tumor cells. Functional analyses demonstrated that LINC00467 silencing decreases migration and invasion *in vitro*. The high expression of LINC00467 is associated with poor distant metastasis-free survival and relapse-free survival (RFS) in patients across different BC subtypes. Also, the expression of LINC00467 negatively correlates with immune and stromal infiltration in BC. Significantly, the copy number amplifications of LINC00467 are related to low infiltration of central and effector memory CD4^+^ and CD8^+^ T cells (**Figure 2**) and are also associated with poor disease-specific survival and progression-free survival in BC patients (85).

### 2.3 LncRNAs related to IL-6 in BC

IL-6 is a pleiotropic cytokine that is crucial in the immune response in non-pathological and pathological conditions. IL-6 can antagonize or promote tumor progression depending on the cell context (86–89). Recent studies indicate that some lncRNAs are related to IL-6 in BC. A study showed that lncRNA BCAR4 is recruited to *PTCH1, MUC5AC, TGF-β1*, and *IL-6* promoters to induce their expression in response to CCL21 in BC cells. Also, the *BCAR4* expression promotes migration and invasion *in vitro* (90). Moreover, DeVaux et al. identified that BHLHE40‐AS1 promotes migration and invasion in ductal infiltrating BC through a low expression of IL-6 and STAT3 phosphorylation (91). Similarly, Nyati et al. found that lncRNA AU021063 expression is promoted by IL-6/Arid5a signaling. Additional analyses showed that AU021063 induces invasion and metastasis of BC *in vitro* and *in vivo* via upregulation of Trib3 and activation of the Mek/Erk signaling pathway (92).

## 3 LncRNAs as tumor-extrinsic regulators of the TIME in BC

Different studies have demonstrated that diverse lncRNAs function as tumor-extrinsic factors specifically expressed by diverse immune cell populations to regulate their functionality, which is essential in the TIME and prognosis in BC. Despite recent advances, the role of lncRNAs as extrinsic regulators of the TIME in BC has been only reported in cytotoxic T lymphocytes, regulatory T cells, and tumor-associated macrophages.

### 3.1 Cytotoxic T lymphocytes

**Figure 3:**
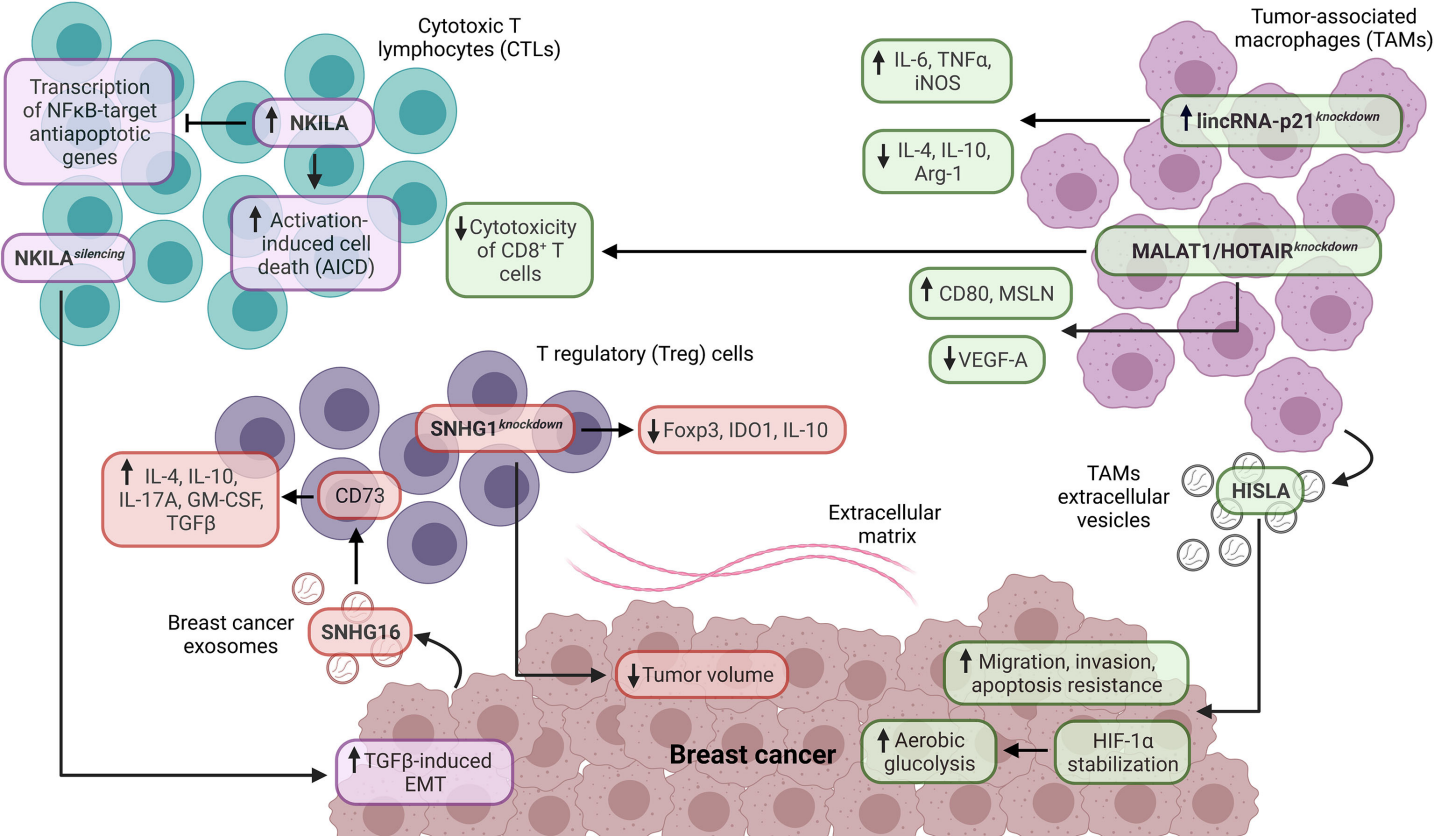
Role of lncRNAs as tumor-extrinsic regulators of the TIME in BC. In the TIME of BC, lncRNAs are critical modulators of the functionality in immune cell populations and BC. In this regard, NKILA is associated with cytotoxic T lymphocytes (CTLs), while SNHG1 is related to regulatory T (Treg) cells. In contrast, lincRNA-p21, HOTAIR, and MALAT1 are related to tumor-associated macrophages (TAMs). In addition, the lncRNAs, such as SNHG16, HISLA, MALAT1, and HOTAIR, are expressed by specific cell populations and have a crucial impact on the functionality of target cell populations in the TIME of BC. EMT, epithelial-mesenchymal transition.

The cytotoxic T lymphocytes (CTLs) are a subpopulation of CD8^+^ T cells that are the main effectors of the antitumoral immune response (24, 93). Remarkably, the CTLs eliminate cancer cells through perforin and granzyme mechanisms. The functionality of CTLs is mainly suppressed in cancer by the induction of anergy states and exhaustion phenotypes (93, 94). Therefore, CTLs are frequently associated with improved prognosis in different cancer types (24). Recent studies have evidenced that lncRNAs are crucial regulators of CTLs in the TIME of BC. Although the role of NKILA in non-neoplastic and neoplastic conditions has been recently reviewed (95), different studies have identified critical roles of this lncRNA in CTLs from the TIME of BC. In this regard, Huang et al. found that NKILA is highly expressed in tumor-specific CTLs and Th1 cells, enhancing their sensibility to activation-induced cell death (AICD) compared to Treg and Th2 cells in BC. Specifically, NKILA suppresses the IκBα phosphorylation, p65 nuclear translocation, and transcription of NFκB-target anti-apoptotic genes in CTLs (96). Additional studies have corroborated the role of NKILA as a negative regulator of NFκB in immune cells from BC (97, 98) (**Figure 3**). Notably, the transcription of NKILA is regulated by calmodulin-induced histone acetylation and STAT1 signaling, and the high levels of NKILA^hi^ tumor-specific CTLs are associated with poor survival in BC patients (96). In contrast, Liu et al. found that the low expression of NKILA is associated with distal metastasis, lymph node status, advanced clinical stage, tumor size, and poor DFS in BC patients (97). Similarly, Wu et al. demonstrated that NKILA silencing promotes TGFβ-induced EMT *in vivo* and the low expression of this lncRNA is associated with poor DFS in BC patients (98) (**Figure 3**). Therefore, NKILA could exert a context-dependent role as a regulator of NFκB signaling and metastasis, suggesting the potential of this lncRNA as a therapeutic target to modulate the function of tumor-specific T cells in BC. In addition, Yu et al. showed that lncRNA expression and CTLs predict the OS and immunotherapy response in cancer patients stratified by immune groups (99).

### 3.2 Regulatory T cells

The regulatory T (Treg) cells are a specialized subpopulation of CD4^+^ T cells characterized by the expression of CD25 and FOXP3. In a non-pathological context, Treg cells suppress the immune response for homeostasis maintaining, self-tolerance, and preventing autoimmune diseases (100). In cancer, Treg cells often promote immunosuppression by expressing IL-10, TGFβ, and CTLA-4. Therefore, Treg cells are frequently associated with poor prognosis in different cancer types (24, 101). Recent research indicates that lncRNAs are important regulators of Treg cells in the TIME of BC. Moallemi-Rad et al. evaluated the Treg cell-related lncRNAs expression from BC in this regard. In particular, RMRP and MAFTRR expression is positively associated with nuclear grade, tubule formation, and tumor size. Conversely, the expression of FLICR differs according to the HER2 levels in BC (102).

SNHG1 and SNHG16 belong to the SNHG lncRNA family, which has a critical oncogenic role in different cancer types (103). Recent findings have evidenced their role as tumor-extrinsic regulators in the TIME of BC. Pei et al. demonstrated that lncRNA SNHG1 is highly expressed on CD4^+^ T cells from the peripheral blood of BC patients, in contrast to CD4^+^ T cells from normal donors. Moreover, the SNHG1 knockdown decreases IDO1, Foxp3, and IL-10 expression, essential Treg cell differentiation promoters. In addition, the SNHG1 knockdown reduces the tumor volume in murine models with BC xenografts (104). In another study, Ni et al. identified that lncRNA SNHG16, delivered from BC exosomes, promotes the activation of the TGF-β1/SMAD5 signaling pathway and miR-16-5p downregulation to induce the upregulation of CD73 in γδ1 Treg cells. The γδ1 Treg cells constitute a high proportion of TIICs in TNBC, ER+PR+, and HER2+ BCs, and the CD73 expression is higher in γδ1 Treg cells derived from BC. Remarkably, CD73^+^ γδ1 Treg cells exhibit high expression of IL-4, IL-10, IL-17A, GM-CSF, and TGFβ, which are critical immunosuppressive molecules, indicating that CD73^+^ γδ1 Treg cells have an immunosuppressive role in the TIME of BC (105) (**Figure 3**).

### 3.3 Tumor-associated macrophages

Tumor-associated macrophages (TAMs) are a crucial cell component in the TIME (106). According to their functions, TAMs are separated into M1 and M2 macrophages. M1 macrophages have pro-inflammatory and antitumoral functions mediated by the secretion of IL-1β, IL-6, IL-12, TNFα, and reactive oxygen species. Moreover, M1 macrophages are key promoters of Th1-type response and are associated with improved prognosis in different cancer types (21, 24, 106). Conversely, M2 macrophages have anti-inflammatory and pro-tumoral functions mediated by the expression of IL-10, TGFβ, PGE2, PD-L1, and PD-L2, promoting immunosuppression. Therefore, M2 macrophages are strongly associated with poor prognosis in cancer patients (21, 24, 106). Interestingly, recent research indicates that lncRNAs are critical regulators of TAMs in the TIME of BC. Zhou et al. evidenced that lincRNA-p21 is upregulated in TAMs from BC murine models. Moreover, the lincRNA-p21 knockdown in TAMs promotes the production of pro-inflammatory molecules (IL-6 and TNFα), iNOS, and decreases the production of anti-inflammatory molecules (IL-4 and IL-10) and Arg-1, indicating that this lncRNA has an essential role in the TAM polarization *in vivo* (**Figure 3**). In addition, the lincRNA-p21 knockdown promotes the interaction of MDM2/p53 to activate the NFκB and STAT3 signaling pathways. Interestingly, the lincRNA-p21 knockdown decreases the BC progression and improves survival *in vivo* (107). On the other hand, Chen et al. found that the extracellular vesicle-packaged lncRNA HISLA from TAMs is delivered to TNBC cells, stabilizing HIF-1α (through the inhibition of PHD2/HIF-1α interaction) and enhancing tumoral aerobic glycolysis, suggesting a metabolic reprogramming of BC mediated by cell-cell communication. Also, HISLA promotes migration, invasion, and apoptosis resistance in BC cells (**Figure 3**). The high expression of this lncRNA is associated with lymph node metastasis and poor DFS in BC patients (108). Another investigation analyzed the relationship between lncRNAs and their immunomodulatory role in TAMs derived from BC. In particular, MALAT1 and HOTAIR are upregulated in TAMs derived from TNBC, HER2+, and hormonal BCs. In these TAMs, the MALAT1 and HOTAIR silencing promotes the upregulation of CD80 and MSLN and the downregulation of VEGF-A. Furthermore, this study showed a cytotoxicity decrease in CD8^+^ T cells against TNBC cells treated with anti-PD-L1 inhibitor and cultured under conditioned media derived from TAMs with MALAT1 and HOTAIR silencing, indicating the role of both lncRNAs as tumor-extrinsic negative modulators of the antitumoral immune response in BC (109) (**Figure 3**).

## 4 LncRNAs as potential biomarkers of the TIME and clinicopathological characteristics in BC

In recent years, several Next-Generation Sequencing (NGS) approaches (i.e. bulk RNA-seq, single cell RNA-seq, whole-genome sequencing and whole-exome sequencing) and bioinformatic tools (i.e., Polysolver, NetMHCpan, CIBERSORTx and MiXCR) have been incorporated for interrogating cancer immunobiology, creating the area known as cancer immunogenomics and immunotranscriptomics (110–114), which includes comprehensive pan-cancer analyses focused on neoantigens prediction (115, 116), MHC class I and class II genes (117, 118), compositional and functional characterization of TIMEs (119, 120), cytolytic activity estimation (121), BCR/TCR repertoires (122) and immunotherapies monitoring (110, 119, 123). Remarkably, large-scale bioinformatics pan-cancer studies have focused on the characterization of lncRNAs as immune-related oncogenic biomarkers and as modifiers of TIMEs, highlighting the potential clinical application of lncRNAs as immunotherapy targets (124, 125). Remarkably, recent findings based on identifying lncRNAs as biomarkers of the TIME and clinicopathological characteristics in BC have gained particular interest. This section highlights relevant studies, primarily based on NGS data mining and bioinformatic approaches, which have explored the role of diverse lncRNAs as individual biomarkers and prognostic models/signatures in the BC immunobiology context.

### 4.1 Individual lncRNAs

Recent advances in BC patients have revealed that individual lncRNAs are promising biomarkers of the TIME and clinicopathological characteristics. A study found that the expression of lncRNA ST7-AS1 is associated with various signaling pathways, including MYC, KRAS, IL6-JAK-STAT3, and apoptosis signaling pathways. In addition, the expression of ST7-AS1 differentially correlates with elevated levels of diverse lymphoid and myeloid cell populations. The high expression of ST7-AS1 is associated with improved OS, progression-free survival, and disease-specific survival (DSS) in BC patients (126).

Yi et al. observed that the expression of SLC26A4-AS1 is associated with the infiltration of diverse immune cell populations. Notably, high expression of this lncRNA is associated with improved OS and DSS in BC patients (127). A study by Zhao et al. revealed that the expression of lncRNA DRAIC is inversely correlated with the infiltration of DCs and neutrophils. High expression of DRAIC is associated with advanced tumor stage, positive lymph node status, and unfavorable OS and DSS in ER+ BC patients (128). Another research demonstrated that elevated expression of lncRNA TCL6 is associated with various immune-related pathways in BC. TCL6 expression positively correlates with infiltration of neutrophils, DCs, B cells, CD4^+^ T, and CD8^+^ T cells, as well as the expression of IC genes, such as *PD-1, PD-L1, PD-L2*, and *CTLA-4*. The low and high expression of TCL6 is associated with poor and improved OS, respectively, in LB BC (129). Similarly, a recent study demonstrated that high and low expression of LINC00426 is consistently associated with increased and poor OS in LB BC, respectively. Moreover, the LINC00426 expression correlates with the infiltration level of diverse immune cell populations, IC, and cytolytic activity-related genes, evidencing that this lncRNA is an immune phenotype-related biomarker in LB BC (130).

Liu et al. reported that lncRNA OSTN-AS1 positively correlates with B and T cell signaling pathways in BC, involving genes like *PDCD1, CTLA-4, CD79A*, and *CD79B*. High expression of OSTN-AS1 is related to diverse immune functions encompassing cytokines, chemokines, NK cells, B cells, T cells, and others. The high expression of OSTN-AS1 is associated with a favorable prognosis in TNBC patients (131). In another study, De Santiago et al. found that LINC00944 is upregulated in TNBC cells due to ADAR1 loss. This lncRNA is related to immune signaling pathways, such as interferon-gamma response, inflammatory response, IL2-STAT5, and TNFα-NFκB. Also, the LINC00944 expression positively correlates with T cell-associated gene markers (*CD3D, CD3E, CD3G, SH2D1A*, and *TRAT1*) in BC patients. Reduced expression of LINC00944 is associated with diminished T-cell infiltration, while the high expression of this lncRNA is related to the upregulation of anti-apoptotic genes. The high expression of LINC00944 is associated with improved RFS in TNBC patients (132). Similarly, a study identified that lncRNAs RP3-460G2.2, RP11-1008C21.1, and RP5-899E9.1 are correlated with the infiltration of diverse immune cell populations and are strongly associated with macrophage gene markers (*CD68* and *MSR1*) in BC (133).

An investigation revealed a negative correlation between LINC00472 expression and IFNγ, IFNα, and TNFα pathways while exhibiting a positive correlation with p53, ER, and PR pathways in ER+, ER- BCs, and TNBC, implying an association with immunosuppression. Conversely, the opposite correlation was detected for lnc-HLA-DRB1-5 in ER+, ER- BCs, and TNBC (134). Notably, recent research has focused on evaluating the expression and roles of immune-related lncRNAs in different BC subtypes. Mathias et al. highlighted the high expression of LINC01871 in BL BC, which participates in interferon-gamma response, allograft rejection, interferon alpha, inflammatory response, IL6-JAK-STAT3, and IL2-STAT5 signaling. Additionally, the upregulation of LINC01871 was associated with improved OS and progression-free interval (PFI) in BL BC (135).

Similarly, XXYLT1-AS2 exhibits high expression in HER2-enriched BC and correlates with improved PFI in this subtype. This lncRNA positively correlates with allograft rejection, interferon-gamma response, inflammatory response, IL-2, and IL-6 signaling, while negatively correlates with EMT, hypoxia, and myogenesis. Conversely, MEG3 is highly expressed in LA BC and positively correlated with TNFα-NFκB, inflammatory response, allograft rejection, interferon-gamma response, and IL2-STAT5 signaling (135). Furthermore, the lncRNA EBLN3P is highly expressed in LB BC and is associated with improved OS. The expression of EBLN3P negatively correlates with TNFα-NFκB and allograft rejection signaling (135).

Recent findings highlighted LINC01087 as a potential promoter of the antitumoral immune response with high expression in luminal BCs. Specifically, LINC01087 demonstrated a relationship with the NFκB signaling pathway in LA and LB BC. Moreover, this lncRNA is also related to chemoattractants, chemokine, and pattern recognition receptors signaling pathways in LA BC. Elevated expression of LINC01087 downregulates oncogenic network components related to proliferation and adhesion, including the WNT/β-catenin pathway. Remarkably, the high expression of LINC01087 was correlated with improved OS and RFS in LA and LB BC patients (136). On the other hand, a recent study analyzed the expression of immune-related lncRNAs in BC, revealing differences based on hormone status. Notably, the low expression of immune-related lncRNAs ENST0000615051, lnc-DDX31, and LINC02381 was detected in ER+ BC, while reduced expression of lnc-DDX31 was observed in PR+ BC (137). Equally important, an investigation found epigenetically dysregulated lncRNAs associated with immune pathways related to inflammation, cytokines, chemokines, and T cells, depending on the BC subtype. In this context, LINC01983, UCA1, RP11-221J22.1 and RP11-221J22.2 were specific to luminal BCs, while RP1-140K8.5, AC005162.1, AC020916.2 SLC26A4-AS1, and CTC-303L1.2 were specific to BL subtype (138). These findings point out the potential of specific lncRNAs as valuable biomarkers for assessing the TIME and predicting clinical outcomes in BC patients.

### 4.2 LncRNA prognostic models/signatures

Recent studies have explored the combined roles of diverse immune-related lncRNAs in models/signatures, which are prognostic predictors and markers of immune landscapes in BC, indicating their potential usefulness in clinical settings (139–143). Liu et al. showed that a nomogram, based on seven immune-related lncRNAs, age, clinical stage, ER status, and BC subtype, is a predictor of OS in BC. Also, the study exhibited that low-risk patients have an enrichment of immune pathways associated with inflammation and a correlation between the infiltration of B cells, T cells, and macrophages with the risk score. In contrast, high-risk patients show a high mutational burden (144). Similarly, a signature based on five immune-related lncRNAs predicts the OS and negatively correlates with the infiltration of B cells, T cells, DCs, neutrophils, and macrophages (145). In this context, different immune-related lncRNA models/signatures are predictors of survival and metastatic status. Notably, the lncRNA models/signatures can stratify BC patients based on their risk score, associated with the enrichment of various immune-related pathways, the abundance of diverse immune cell populations, and the expression of different IC genes (**Table 1**).

**Table 1:** Immune-related lncRNA models/signatures are prognostic predictors and markers of the TIME characteristics in BC.

Number of lncRNAs in the model/signature	Prognostic value in patients	TIME characteristics determined by the model/signature	Reference
4	MFS, OS	A high RS is associated with aDCs, eosinophils, immature B cells, pDCs, Treg cells, and T_H_2 cells. A low RS is associated with CD56dim NK cells, monocytes, and neutrophils.	(146)
4	RFS	The signature is associated with leukocytes, lymphocytes, B cells, T cells, cytokines, IFNγ production, antigen receptor, and regulation of different immune and intracellular processes-related pathways.	(147)
5	OS	The lncRNAs negatively correlate with CD4 T cells, CD8 T cells, B cells, DCs, neutrophils, and macrophages.	(145)
5	Metastatic status	The signature correlates with gene markers associated with B cells, naive T cells, effector T cells, resident memory T cells, T_H_1 cells, Treg cells, T cell exhaustion, macrophages, TAMs, monocytes, NK cells, neutrophils, and DCs.	(148)
6	OS	A high RS is associated with a low enrichment of CD8 T cells and with dysregulations in the endoplasmic reticulum and antigen processing and presentation pathways. A low RS is associated with high enrichment of CD8 T cells.	(149)
7	OS	The RS negatively correlates with CD8 T cells, resting memory CD4 T cells, naive B cells, and memory B cells. Also, the RS positively correlates with M0 and M2 macrophages. A low RS is associated with the enrichment of IFN response pathways-related genes.	(144)
8	OS	A low RS is associated with the enrichment of positive regulation of immune effector processes, positive regulation of adaptive immune response, positive regulation of lymphocyte activation, regulation of T cell activation, and T cell receptor signaling pathways.	(150)
10	OS, tumor mutational burden, immunotherapy response	A low RS is enriched with different immune-related functions, infiltration of diverse immune cell populations, reduced expression of immune checkpoint genes, poor OS, and high TIDE score compared to the high RS group.	(151)
11	OS	The RS negatively correlates with B cells, CD4^+^ T cells, CD8^+^ T cells, DCs, neutrophils, and macrophages.	(152)
13	OS	A high RS positively correlates with plasma cells, M2 macrophages, neutrophils, and low expression of PD-L1. A low RS positively correlates with the infiltration of diverse immune cell populations.	(153)
40	OS	A high RS positively correlates with M0 and M2 macrophages and low expression of *LAG3, CTLA-4, PDCD1*, and *PDCD1LG2*. Also, the high RS negatively correlates with CD8^+^ T cells.	(154)
56	OS	A low RS is associated with high infiltration of aDCs, B cells, CD8 T cells, DCs, NK CD56dim cells, NK CD56bright cells, pDCs, Tfh, T_H_17, T_H_2, and Treg cells. Moreover, a low RS is associated with low infiltration of macrophages, Tem, Tcm, Tgd, and T_H_1 cells.	(155)

aDCs, activated dendritic cells; CD, cluster of differentiation; DCs, dendritic cells; IFN, interferon; MFS, metastasis-free survival; NK, natural killer; OS, overall survival; pDCs, plasmacytoid dendritic cells; RFS, relapse-free survival; RS, risk score; TAMs, tumor-associated macrophages; Tcm, T central memory cells; Tem, T effector memory cells; Tfh, T follicular helper cells; Tgd, T gamma-delta cells; TH, T helper cells; TIDE, tumor immune dysfunction and exclusion; TIME, tumor immune microenvironment; Treg, regulatory T cells.

Interestingly, different studies have demonstrated a relationship between tumor immune response and ferroptosis, necroptosis, pyroptosis, autophagy, and genomic instability processes (156–161). In this regard, various studies have developed prognostic lncRNA models/signatures related to these processes, which predict the TIME characteristics and immunotherapy response in BC patients (**Table 2**). Additional lncRNA prognostic models/signatures focused on other biological processes, such as lipid metabolism, hypoxia, glycolysis, EMT, stemness, RNA-binding proteins, endoplasmic reticulum stress, cuproptosis, lactate, oxidative stress, androgen receptor signaling pathway, mitochondrial function, aging and angiogenesis have also demonstrated to be predictors of immune landscapes characteristics in BC (176–190). However, further studies are mandatory to explore the relationship between these processes with BC immunobiology.

**Table 2:** LncRNA models/signatures related to ferroptosis, necroptosis, pyroptosis, autophagy, and genomic instability are prognostic predictors and markers of the TIME characteristics in BC.

Number of lncRNAs in the model/signature	Prognostic value in patients	TIME characteristics determined by the model/signature	Reference
5 (ferroptosis-related)	RFS	Patients in the high-risk group have low expression of ICs.	(162)
7 (ferroptosis-related)	OS	A high RS is associated with low infiltration of macrophages, DCs, CD8^+^ T, and B cells.	(163)
8 (ferroptosis-related)	DSS, OS, PFS	Low-risk patients have an enrichment of antigen processing and presentation, NK cell-mediated cytotoxicity, T cell receptor, and chemokine signaling pathways. Also, these patients have a high proportion of tumor-infiltrating CD8^+^ T cells, activated NK cells, and M1 macrophages and high expression of PD1, PDL1, CTLA-4, LAG3, and TIGIT.	(164)
10 (ferroptosis-related)	OS	Patients in the low-risk group have an enrichment of diverse immune-related processes.	(165)
11 (ferroptosis-related)	OS	A low RS is associated with the enrichment of NK, T, and B cells. A high RS is associated with the enrichment of M1 macrophages and cancer-associated fibroblasts. Patients in the high-risk group have an enrichment of ICs.	(166)
4 (necroptosis-related)	OS	A high RS is negatively associated with infiltration of memory B cells, activated memory CD4^+^ T cells, CD8^+^ T cells, aDCs, M0 and M1 macrophages, activated NK cells, plasma cells, Tfh, Treg cells, and positively associated with infiltration of M2 macrophages, resting memory CD4^+^ T cells, resting DCs, resting mast cells, naive B cells, and eosinophils. A low RS is associated with high expression of 36 ICs, and with enrichment of cell cycle, cytokine-cytokine receptor interaction, chemokine signaling pathway, primary immunodeficiency, and T cell receptor signaling pathway.	(167)
7 (necroptosis-related)	Immunotherapy response	The RS positively correlates with aDCs, M0, and M2 macrophages. Low-risk patients have a high stromal and immune infiltration score and a high TIDE score.	(168)
13 (necroptosis-related)	OS, immunotherapy response	A low RS is associated with high infiltration of naive B cells, monocytes, activated NK cells, plasma cells, CD4^+^ activated memory T cells, and CD8^+^ T cells. Patients in the high-risk group have high infiltration of M0 and M2 macrophages and neutrophils. Patients in the low-risk group have an enrichment of ICs like PD-L1, CD28, and CTLA-4. Also, these patients have an enrichment of diverse immune-related processes.	(169)
5 (autophagy-related)	OS	Patients in the low-risk group have an enrichment of antigen processing and presentation and T cell receptor pathways.	(170)
11 (autophagy-related)	OS	A high RS correlates with infiltration of central memory CD8 T cells, Tfh cells, and memory B cells. A low RS correlates with infiltration of activated CD8 T cells, effector memory CD8 T cells, T_H_1 cells, activated B cells, immature B cells, NK cells, eosinophils, mast cells, and monocytes.	(171)
8 (pyroptosis-related)	OS, immunotherapy response	Patients in the high-risk group have a low abundance of immune infiltrating cell populations and have inhibition of antigen processing and presentation, apoptosis, B-cell receptor, T-cell receptor, and JAK-STAT pathways. Responding patients have a low RS than non-responding patients to anti-PD-1 treatment.	(172)
10 (pyroptosis-related)	OS	A low RS is associated with a high abundance of CD8^+^ T cells, activated memory CD4^+^ T cells, B cells, and NK cells. Patients in the high-risk group have infiltration of Treg cells and M2 macrophages. Patients in the low-risk group have high expression of T cell phenotypic and functional markers, activating immune receptors, IFNγ signature, and IC markers.	(173)
7 (genomic instability-related)	OS, number of somatic mutations	Patients in the high-risk group have low levels of CD8^+^ T cells and increased levels of M2 macrophages. Moreover, in this group, the CXCL8 expression is positively correlated with M2 macrophages and negatively correlated with CD8 T cells.	(174)
128 (genomic instability-related)	OS, number of somatic mutations	A high RS is positively associated with high expression of negative ICs (CTLA-4, CD276, TIGIT, PVR, HMGB1, TDO2, IDO1, CXCL9, and CXCL10). A low RS is positively associated with the expression of positive ICs (TNFRSF9, TNFRSF14, and TNFRSF18).	(175)

aDCs, activated dendritic cells; CD, cluster of differentiation; DCs, dendritic cells; DSS, disease-specific survival; IC, immune-checkpoint; IFN, interferon; MFS, metastasis-free survival; NK, natural killer; OS, overall survival; PFS, progression-free survival; RFS, relapse-free survival; RS, risk score; Tfh, T follicular helper cells; TH, T helper cells; TIDE, tumor immune dysfunction and exclusion; TIME, tumor immune microenvironment; Treg, regulatory T cells.

## 5 Potential limitations and advantages of lncRNAs for BC immunotherapy

Diverse studies have demonstrated the importance of lncRNAs in cancer biology (38–42). As discussed in this review, lncRNAs are critical players in diverse BC-intrinsic and extrinsic immune-related processes. Also, lncRNAs are potential biomarkers of patients’ TIME and clinicopathological characteristics. Previous studies have supported the potential usefulness of strategies for targeting lncRNAs in a cancer context. These approaches are mainly focused on post-transcriptional targeting [i.e., RNA-mediated interference (RNAi), Morpholino oligomers, and anti-sense oligonucleotides (ASOs)], modulation of lncRNA-expressing loci via CRISPR/Cas9-based genome editing, transcriptional upregulation through targeting natural anti-sense RNAs, steric inhibition of lncRNA function, and lncRNA tertiary structure disrupting-based strategies (43, 44, 191–193). Notably, a comprehensive study used ASOs to target LINC00624 in HER2+ BC, resulting in the inhibition of proliferation *in vitro* and increasing the expression of innate immune response-related genes in xenograft tumor models, supporting the role of LINC00624 as an inhibitor of the antitumoral immune response (76). In addition, a CRISPR activation screening strategy was recently used to determine the mechanistic role of LINC01198 and IL10RB-DT in BC cells, concluding an association with promoting and suppressing the antitumoral immune response, respectively (67). These findings highlight lncRNAs as attractive targets for BC immunotherapy. However, some issues must be addressed before incorporating lncRNAs in clinical settings. Firstly, most of the functional studies focused on lncRNAs, in the context of BC immunobiology, are based on targeting strategies, like CRISPR-Cas9, RNAi and ASOs, and routine functional assays *in vitro* and *vivo* (i.e., co-cultures, proliferation, migration and invasion assays). Despite these advances, there is still a gap in our understanding of the exact mechanistic role of lncRNAs; therefore, the incorporation of comprehensive functional approaches and complementary strategies to fully dissect the crosstalk of lncRNAs in signaling pathways, lncRNA tertiary structure, and lncRNAs interactions with diverse RNA species, DNA elements, chromatin, and proteins are mandatory to completely understand the versatile and complex mechanistic roles of lncRNAs in BC immunobiology. Secondly, bioinformatic studies focused on lncRNAs must be validated using experimental methodologies like flow cytometry and multiplex immunofluorescence. Thirdly, we still lack information about the tumor extrinsic roles of diverse lncRNAs in the remaining BC TIME cell components, such as B cells, MDSCs, and neutrophils. Additionally, clinical trials by FDA and EMA are mandatory for validating immune-related prognostic biomarkers and immunotherapy strategies based on lncRNAs in BC, considering the current hurdles associated with non-coding RNA therapeutics, such as on-target specificity, unwanted off-target effects, and delivery systems (43, 44, 192, 194).

Despite these challenges, lncRNAs are promising molecules for BC immunotherapy because different molecules like XIST, LINC001198, TINCR, LINK-A, and HEIH function as promoters or suppressors of the antitumoral immune response at intrinsic and extrinsic levels, demonstrated by *in vivo* and *in vitro* studies. Also, investigations based on NGS data mining from public repositories and bioinformatic analyses have elucidated the role of diverse lncRNAs like DRAIC, OSTN-AS1, LINC00944, and LINC01871 as biomarkers of the TIME and clinicopathological features in BC, highlighting lncRNAs as potential immunotherapy targets. In addition, lncRNA targeting strategies may be combined with current and approved immunotherapies based on protein and cellular targets (i.e., IC inhibitors, cytokine modulation, and immune cell-based therapies) to increase the therapeutic options, improve the response to immunotherapies and consider personalized treatments for BC patients. Also, lncRNAs like GATA3-AS1, LINC00624, TCL6, LINC00426, and MIAT have BC subtype-specific expression that can be useful for proper designing and specific implementation for patients’ stratification strategies and immunotherapies based on lncRNAs in BC in the next coming years.

## 6 Conclusion and perspectives

The dysregulation of lncRNAs has a crucial role in tumorigenesis and cancer progression. Mainly, various studies have reported the relevance of different lncRNAs in alterations of processes associated with cancer immunobiology. In this review, we provided the current advances in the role of lncRNAs as modulators of antitumoral immune response and the TIME in BC, as well as their role as potential biomarkers of the TIME and clinicopathological characteristics in BC patients. The pivotal role of lncRNAs in regulating antigen processing and presentation, ICs expression, infiltration, and functionality of immune cell populations, and their association with diverse prognosis parameters, highlights that lncRNAs are potential biomarkers of immune phenotypes and immunotherapy targets for BC. Limitations in our knowledge of lncRNAs in BC immunobiology are associated with the complexity of thoroughly dissecting their exact mechanistic roles and interactions. Therefore, future lncRNA research based on comprehensive functional strategies, bioinformatics approaches, and clinical trials is mandatory to fully understand the versatile and complex mechanistic and clinical roles of diverse lncRNAs in BC immunobiology. Taken together, the advances in lncRNAs have opened a novel and exciting area to dissect BC immunobiology and its potential therapeutic significance in the next coming years.

## Author contributions

Conceptualization, investigation, resources, original draft, figures and tables preparation: MAF-M. Writing and editing: MAF-M. Review and comments: KIV-S. Supervision: AH-M. All authors contributed to the article and approved the submitted version.
